# Angiopoietin-1 inhibits tumour growth and ascites formation in a murine model of peritoneal carcinomatosis

**DOI:** 10.1038/sj.bjc.6600598

**Published:** 2002-11-04

**Authors:** O Stoeltzing, S A Ahmad, W Liu, M F McCarty, A A Parikh, F Fan, N Reinmuth, C D Bucana, L M Ellis

**Affiliations:** Department of Cancer Biology, The University of Texas M.D. Anderson Cancer Center, Houston, Texas, TX 77030, USA; Department of Surgical Oncology, The University of Texas M.D. Anderson Cancer Center, Houston, Texas, TX 77030, USA

**Keywords:** angiopoietins, angiogenesis, vascular permeability, ascites, colon cancer

## Abstract

Angiopoietin-1 is an important regulator of endothelial cell survival. Angiopoietin-1 also reduces vascular permeability mediated by vascular endothelial growth factor. The effects of angiopoietin-1 on tumour growth and angiogenesis are controversial. We hypothesised that angiopoietin-1 would decrease tumour growth and ascites formation in peritoneal carcinomatosis. Human colon cancer cells (KM12L4) were transfected with vector (pcDNA) alone (control) or vector containing angiopoietin-1 and injected into the peritoneal cavities of mice. After 30 days, the following parameters were measured: number of peritoneal nodules, ascites volume, and diameter of the largest tumour. Effects of angiopoietin-1 on vascular permeability were investigated using an intradermal Miles assay with conditioned media from transfected cells. Seven of the nine mice in the pcDNA group developed ascites (1.3±0.5 ml (mean±s.e.m.)), whereas no ascites was detectable in the angiopoietin-1 group (0 out of 10) (*P*<0.01). Number of peritoneal metastases (*P*<0.05), tumour volume, (*P*<0.05), vessel counts (*P*<0.01), and tumour cell proliferation (*P*<0.01) were significantly reduced in angiopoietin-1-expressing tumours. Conditioned medium from angiopoietin-1-transfected cells decreased vascular permeability more than did conditioned medium from control cells (*P*<0.05). Our results suggest that angiopoietin-1 is an important mediator of angiogenesis and vascular permeability and thus could theoretically serve as an anti-neoplastic agent for patients with carcinomatosis from colorectal cancer.

*British Journal of Cancer* (2002) **87**, 1182–1187. doi:10.1038/sj.bjc.6600598 www.bjcancer.com

© 2002 Cancer Research UK

## 

Angiogenesis has been associated with aggressive disease in human colorectal cancer (Takahashi *et al*, 1995, 1998). Although the vascular endothelial growth factor (VEGF) receptor-ligand system has been implicated as a critical mediator of vasculogenesis and angiogenesis, the angiopoietins (Ang-1 to Ang-4) have also been shown to be important regulators of neovascularisation and endothelial cell survival in malignant and non-malignant tissues (Peters, 1998). Ang-1 has been recognised as the major ligand that activates the tyrosine kinase receptor Tie2, thereby promoting endothelial cell survival and vessel stabilisation by recruiting and sustaining peri-endothelial supporting cells (Fujikawa *et al*, 1999; Hayes *et al*, 1999; Kwak *et al*, 1999; Papapetropoulos *et al*, 1999). Ang-2 is the naturally occurring antagonist to Ang-1; Ang-2 prevents Tie2 activation and results in vessel destabilisation (Maisonpierre *et al*, 1997). Studies in transgenic mice revealed that inactivation of Ang-1 or the Tie2 receptor causes embryos to die from defects in vascular remodelling (Dumont *et al*, 1994; Sato *et al*, 1995; Suri *et al*, 1996). Recently, studies have shown that the angiopoietins are sequentially expressed during the angiogenic process, indicating that they are also important modulators of post-natal neovascularisation. Balanced and sequential expression of the angiopoietins and VEGF is required for successful angiogenesis (Asahara *et al*, 1998; Ray *et al*, 2000). In addition, Ang-1 has been shown to override VEGF-mediated effects on vascular permeability (vessel leakage) of adult vasculature (Thurston *et al*, 1999, 2000).

The effects of the angiopoietins on angiogenesis, tumour growth and vascular permeability remain controversial (Suri *et al*, 1998; Chae *et al*, 2000; Hayes *et al*, 2000; Ahmad *et al*, 2001; Shim *et al*, 2001). Some studies suggest that Ang-1 may be pro-angiogenic (Suri *et al*, 1998; Hansbury *et al*, 2001; Shim *et al*, 2001), whereas others have shown that Ang-1 inhibits angiogenesis, tumour growth and vascular permeability (Chae *et al*, 2000; Hayes *et al*, 2000; Thurston *et al*, 2000; Ahmad *et al*, 2001; Tian *et al*, 2002).

We hypothesised that overexpression of Ang-1 by human colon cancer cells would decrease angiogenesis, tumour growth and metastasis formation and inhibit ascites formation in an experimental model of peritoneal carcinomatosis. Our results suggest that Ang-1 is an important regulator of angiogenesis and vascular permeability and thus indicate that Ang-1 could theoretically serve as an anti-angiogenic agent in the treatment of metastatic colorectal cancer.

## MATERIALS AND METHODS

### Cell culture and transfection

The highly metastatic human colon cancer cell line KM12L4 was obtained from IJ Fidler (The University of Texas M.D. Anderson Cancer Center) (Morikawa *et al*, 1988). KM12L4 cells were cultured and maintained on plastic in modified Eagle's medium supplemented with 10% foetal bovine serum, 2 units ml^−1^ penicillin–streptomycin mixture (Flow Laboratories, Rockville, MD, USA), two-fold vitamin solution (Life Technologies, Inc., Grand Island, NY, USA), 1 mM sodium pyruvate, 2 mM L-glutamine and non-essential amino acids and incubated in 5% CO_2_–95% air at 37°C as previously described (Shaheen *et al*, 2001).

The full-length cDNA for Ang-1 was provided by Tona Gilmer (GlaxoSmithKline, Research Triangle Park, NC, USA). The construct was sub-cloned into a pcDNA3 vector (InVitrogen, Carlsbad, CA, USA) containing a hygromycin resistance gene (Ahmad *et al*, 2001). Vector alone or vector containing Ang-1 was transfected into KM12L4 cells using Lipofectin according to the manufacturer's protocol (Boehringer Mannheim Co., Randburg, South Africa), and the transfected cells were grown in selective media as previously described (Ahmad *et al*, 2001). Successful transfection of Ang-1 was confirmed by Northern blot analysis as previously described (Ahmad *et al*, 2001).

For *in vivo* studies, Ang-1- and pcDNA-transfected KM12L4 cells were harvested from sub-confluent cultures. Briefly, cells were rinsed with phosphate-buffered saline (PBS), trypsinised (0.25% trypsin; 0.02% EDTA) and re-suspended in 10% foetal bovine serum–modified Eagle's medium. Cells were counted and cell viability was assessed using the Trypan blue dye exclusion assay (>90% viability). Cells were resuspended in Hank's balanced salt solution for tumour cell inoculation into mice.

### *In vivo* studies

Eight-week-old male athymic nude mice (obtained from the Animal Production Area of the National Cancer Institute and Development Center, Frederick, MD, USA) were acclimated for 1–2 weeks while caged in groups of five. Mice were housed as previously described (Shaheen *et al*, 2001) and fed a diet of animal chow and water *ad libitum* throughout the experiment. Experiments were approved by the Animal Care and Use Committee at M. D. Anderson Cancer Center and met all the standards required by the UKCCCR guidelines for the welfare of animals in experimental neoplasia, as published (United Kingdom Co-Ordinating Committee on Cancer Research, (1998)).

#### Peritoneal carcinomatosis model

Mice were randomly assigned to one of two groups (*n*=10 per group). The average body weight among groups was similar at assignment. Under sterile conditions, either Ang-1- or pcDNA (control)-transfected KM12L4 cells (1×10^6^ cells) were injected into the peritoneal cavity of each mouse by means of a 30-gauge needle. An injection volume of 500 μl (in Hank's balanced salt solution) was used to achieve a distribution adequate for peritoneal implantation of tumour cells. Animals were monitored daily (weighed once per week), and when mice in any group showed early signs of decreased mobility, all animals were killed by cervical dislocation after anaesthesia induction with pentobarbital (Nembutal, 50 mg kg^−1^). Body weight was determined, and ascites was collected by performing a lower midline incision and completely draining the intra-abdominal fluid into a flask. The ascites volume was then measured with a 1-ml tuberculin syringe. Incisions were then extended to allow photography of the peritoneal cavity and determination of the extent of peritoneal carcinomatosis. Peritoneal tumour nodules were counted, and the diameter of the largest tumour (always occurring in the greater omentum) was determined. Tumour tissue was then harvested, placed in either 10% formalin for paraffin embedding or snap-frozen in optimum cutting temperature solution (Miles Inc., Elkhart, IN, USA) in preparation for subsequent immunohistochemical analyses.

#### Intradermal Miles assay

To investigate the effects of overexpression of Ang-1 by tumour cells on vascular permeability, the intradermal Miles assay was performed as described previously (Zebrowski *et al*, 1999). Conditioned medium (CM) from Ang-1- and pcDNA-transfected KM12L4 cells was collected after a 48-h incubation in 5% foetal bovine serum–modified Eagle's medium at 80% cell density, centrifuged for 5 min at 2000 r.p.m. and filtered through a 0.22-μm filter. Mice (*n*=3 per group) were injected intravenously with sterile 0.5% Evans blue dye (200 μl) via the tail vein. Ten minutes later, mice were given four separate intradermal injections at different sites into the dorsal skin – one each of CM-Ang-1, CM-pcDNA, PBS (negative control), and PBS plus VEGF (10 ng ml^−1^) (R&D Systems Inc., Minneapolis, MN, USA) (positive control for increased vascular permeability). The injections were done using a 30-gauge needle, and the injection volume was 50 μl. Mice were sacrificed 20 min after intradermal injections by cervical dislocation following anaesthesia induction with nembutal. The dorsal skin of each mouse was harvested to permit visualisation of intradermal dye leakage. To determine the relative degree of vascular permeability, two dimensions (*a* and *b*) of the elliptically appearing area of dye leakage were obtained at each injection site and the area was calculated by the formula *a*×*b*×π. Densitometric analysis was performed using the NIH Image Analysis software (V1.62) from the National Institute of Health (Bethesda, MD, USA) as another means of quantifying the extent of dye leakage at the intradermal injection sites in each mouse. Digitally obtained images of the underside of the dorsal skin, including all injection sites, were converted to a gray-scale image, and dye density was analysed at each site (threshold was set individually for each dorsal skin flap, but was constant for each mouse).

### Immunohistochemical analyses of tumour vessel density

Antibodies for immunohistochemical analyses were obtained as follows: rat anti-mouse CD31 antibody from PharMingen (San Diego, CA, USA) and peroxidase-conjugated goat anti-rat IgG from Jackson Research Laboratories (West Grove, PA, USA). Tumours that had been frozen in optimum cutting temperature solution were sectioned at 8-μm intervals, mounted on positively charged slides and air-dried for 30 min. Tissue sections were then fixed in cold acetone, followed by 1 : 1 acetone/chloroform and acetone, and then washed with PBS. Specimens were then incubated with 3% H_2_O_2_ in methanol for 12 min at room temperature to block endogenous peroxidase, washed with PBS (pH 7.5) and incubated for 20 min at room temperature in a protein-blocking solution consisting of PBS supplemented with 1% normal goat serum and 5% normal horse serum. The primary antibody directed against CD31 was diluted 1 : 800 in protein-blocking solution and applied to the sections, which were incubated overnight at 4°C. Sections were then rinsed in PBS and incubated for 10 min in protein-blocking solution before the addition of peroxidase-conjugated secondary antibody. The secondary antibody used for CD31 staining (peroxidase-conjugated goat anti-rat IgG) was diluted 1 : 200 in protein-blocking solution. After incubation with the secondary antibody for 1 h at room temperature, the samples were washed and incubated with stable diaminobenzidine substrate (Research Genetics, Huntsville, AL, USA). Staining was monitored under a bright-field microscope, and the reaction was stopped by washing with distilled water. Sections were counterstained with Gill No. 3 haematoxylin solution (Sigma Chemical Co., St. Louis, MO, USA) and mounted with Universal Mount (Research Genetics). CD31-stained vessels were counted at 50× magnification at four different quadrants of each tumour (2 mm inside the tumour–normal tissue interface), and average vessel counts were calculated. For all immunohistochemical procedures, the primary antibody was omitted as a negative control.

### Immunohistochemical analyses of tumour cell proliferation

Paraffin-embedded tumours were sectioned at 4- to 6-μm intervals, mounted on positively charged Superfrost slides (Fisher Scientific Co., Houston, TX, USA) and allowed to dry overnight at room temperature. Sections were deparaffinised in xylene, treated with a graded series of alcohol washes (100, 95, and 80% ethanol in double-distilled H_2_O (v v^−1^)), rehydrated in PBS (pH 7.5) and microwaved for 5 min for antigen retrieval. Tumour sections were stained for proliferating cell nuclear antigen (PCNA) using mouse anti-PCNA clone PC10 DAKO A/S (DAKO Corp., Carpinteria, CA, USA). Immunohistochemical procedures were performed as described previously (Ahmad *et al*, 2001). Positive reactions were visualised by incubating the slides with stable diaminobenzidine for 10–20 min. The sections were rinsed with distilled water, counterstained with Gill No. 3 haematoxylin solution for 1 min, and mounted with Universal Mount. The number of tumour cells staining and not staining for PCNA was determined (at 100× magnification) at four staining ‘hot spots’ per tumour sample, and the percentage of PCNA-positive cells was then calculated. Necrotic areas of the tumour were excluded. Slides were also stained with haematoxylin and eosin to permit study of overall tissue morphology.

### Statistical analyses

All statistical analyses were done using InStat Statistical Software version 2.03 (GraphPad Software, San Diego, CA, USA), with *P* values less than 0.05 considered to be statistically significant. Tumour-associated parameters were tested for statistical significance using the two-sided Student's *t*-test or Mann–Whitney *U*-test (for non-parametric data). Fisher's exact test was applied for comparing incidences of peritoneal carcinomatosis and ascites formation.

## RESULTS

### Effect of angiopoietin-1 expression on formation of peritoneal metastases and tumour growth

To study the effect of Ang-1 on peritoneal implantation and growth of human colon cancer cells, pcDNA- and Ang-1-transfected KM12L4 cells were injected into the peritoneal cavities of nude mice. Thirty days after tumour cell inoculation, three of the control (pcDNA) mice showed decreased mobility (secondary to ascites), and the experiment was terminated. (One mouse in the pcDNA group died at day 21 of acute bowel obstruction caused by a peritoneal tumour). Peritoneal carcinomatosis developed in seven (78%) of the nine mice in the control group, compared to three (30%) of the 10 mice of the Ang-1 group. However, the incidences of development of carcinomatosis were not significantly different (*P*=0.07).

**Figure 1:**
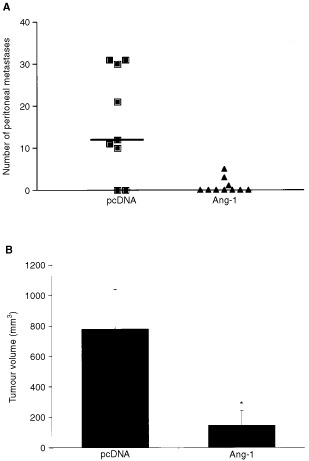
Effect of Ang-1 overexpression on tumour growth. Ang-1- or pcDNA-transfected KM12L4 colon cancer cells were injected into the peritoneal cavities of mice. (**A**) After 30 days, mice were killed and peritoneal metastases were counted. Overexpression of Ang-1 in colon cancer cells significantly reduced the number of peritoneal metastases (*P*<0.01, Mann–Whitney *U*-test) compared to controls (pcDNA). Bars: median. (**B**) The largest tumour (greater omentum) was measured in each mouse. Tumour volumes in the Ang-1 group were significantly smaller than tumours in the pcDNA group (*P*<0.05, Mann–Whitney *U*-test). Bars: s.e.m.

To evaluate the effects of Ang-1 overexpression on tumour burden in mice, the number of peritoneal metastases and the volume of the largest tumour (occurring in the greater omentum) were calculated for each mouse. The total number of peritoneal metastases was significantly lower in the Ang-1 group than in the control group (*P*<0.01) (Figure 1A

). Peritoneal nodules were additionally categorised into size ranges (greatest diameter), as a more precise volume measurement (cubic mm) was not feasible due to their abundance. Small tumours (1–5 mm) were detectable at frequency of 2.2±1.4 nodules/mouse (mean ±s.e.m.)) in the pcDNA group and a mean of 0.3±0.2 nodules/mouse in the Ang-1 group (*P*<0.05). Large tumours (6–15 mm) were detectable at frequency of 13.4±3.3 nodules/mouse in the pcDNA group and compared to 0.5±0.3 nodules/mouse in the Ang-1 group (0.5±0.3) (*P*<0.05). As tumour growth beyond 1–2 mm is angiogenesis dependent, measurement of the largest tumour was a consistent and unbiased reflection of the effects of Ang-1 on tumour angiogenesis. The maximal tumour volume was significantly less in the Ang-1 group than in the pcDNA group (*P*<0.05) (Figure 1B).

### Effect of angiopoietin-1 expression on ascites formation

**Figure 2:**
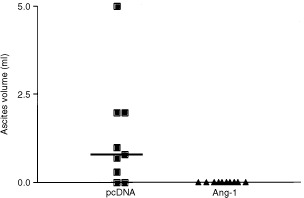
Impact of Ang-1 expression on ascites formation in peritoneal carcinomatosis. Thirty days after tumour cell inoculation, ascites volumes were measured in each mouse. Seven of the nine mice in the pcDNA group developed detectable ascites, with a median ascites volume of 1.3 ml. In contrast, none of the 10 mice in the Ang-1 group developed ascites (*P*<0.01, Fisher's Exact test). Bars: median.

The incidence of ascites formation was significantly higher in mice injected with pcDNA-transfected KM12L4 cells (7 out of 9, 78%) than in mice injected with Ang-1-overexpressing KM12L4 cells (0 out of 10) (*P*<0.01, Fisher's exact test). The ascites volumes in peritoneal metastases–bearing mice in the pcDNA group ranged from 0.3 ml to 5 ml, with a median ascites volume of 1.3 ml (haemorrhagic ascites in all cases). The median ascites volume was significantly higher in the pcDNA group (*P*<0.01) than in the Ang-1 group as none of the Ang-1 mice developed ascites even though three of them had peritoneal tumours (Figure 2

).

### Effect of angiopoietin-1 expression on microvessel density and tumour cell proliferation

**Figure 3:**
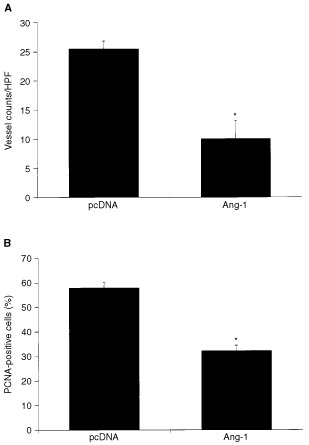
Effect of Ang-1 overexpression on tumour vessel density and tumour cell proliferation. (**A**) Tumour vessels were stained for CD31, and vessel counts were performed as described in Materials and Methods. Vessel counts in Ang-1 tumours were significantly lower than vessel counts in pcDNA tumours (*P<0.01, two-sided Student's *t*-test). (**B**) In addition, the percentage of proliferating tumour cells identified by PCNA staining was significantly reduced in Ang-1-overexpressing tumours (*P<0.01, two-sided Student's *t*-test). Bars: s.e.m. HPF, high-power field.

To determine the effects of Ang-1 overexpression on tumour neovascularisation and tumour cell proliferation, peritoneal metastases were sectioned and evaluated by immunohistochemical analyses. Microvessel density was significantly reduced in Ang-1-transfected KM12L4 tumours relative to controls (pcDNA) (*P*<0.01) (Figures 3A

and 4

**Figure 4:**
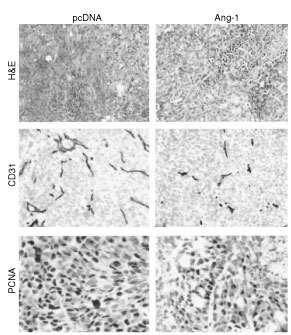
Immunohistochemical analysis of tumour vessel density (CD31) and tumour cell proliferation (PCNA) in peritoneal tumours. Tumour sections were stained with haematoxylin and eosin (H&E), anti-CD31 antibody or PCNA antibody as described in Materials and Methods. Images were obtained at 50× (H&E, CD31) or 100× (PCNA) magnification.

). In addition, analysis of PCNA immunohistochemical staining revealed that Ang-1-overexpressing tumours had a significantly lower percentage of proliferating tumour cells in peritoneal metastases compared to control tumours (*P*<0.01) (Figures 3B and 4).

### Effect of angiopoietin-1 expression on vascular permeability

**Figure 5:**
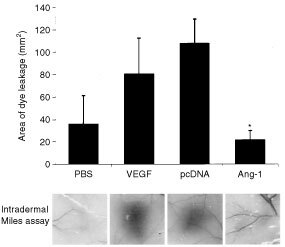
Effect of Ang-1 overexpression by tumour cells on dermal vascular permeability. Effects on vascular permeability were examined using the Miles *in vivo* permeability assay. CM from Ang-1- or pcDNA-transfected cells was injected into the dermis of mice. After 20 min, mice were killed, and the areas of dye leakage (as a measure of permeability) were calculated for each injection site. PBS and PBS plus VEGF (10 ng ml^−1^) served as negative and positive controls, respectively. In contrast to CM of pcDNA-transfected cells, Ang-1-containing CM did not increase plasma leakage, indicating that Ang-1 abrogated permeability effects of tumour cell–derived growth factors in CM (*P<0.05, two-sided Student *t*-test). Bars: s.e.m.

To demonstrate whether the observed inhibition of ascites formation was attributable to reduced vascular permeability mediated by Ang-1 and not solely dependent on tumour burden, we used the Miles *in vivo* permeability assay to determine the direct effect of Ang-1 on vascular permeability. In this assay, effects on vascular permeability were investigated using conditioned media from Ang-1- or pcDNA-transfected KM12L4 cells. Injections with PBS and PBS plus VEGF served as a negative and positive control, respectively. Areas of intradermal dye leakage (a measure of vascular permeability) were significantly smaller at CM-Ang-1 injection sites than at CM-pcDNA injection sites (or PBS-plus-VEGF injection sites, the positive control) (*P*<0.05) (Figure 5

). All injection sites were additionally analysed by densitometry to verify these results. Dye density at CM-Ang-1 sites (39.4±8.2 pixels^2^×10^3^ (mean±s.e.m.)) was significantly lower compared to CM-pcDNA sites (76.8±2.9 pixels^2^×10^3^) (*P*<0.01, Student's *t*-test).

## DISCUSSION

In the present study, overexpression of Ang-1 by human colon cancer cells inhibited tumour angiogenesis, growth of peritoneal metastases and ascites formation in an experimental model of peritoneal carcinomatosis. In studies using the HT29 human colon cancer cell line transfected with Ang-1, similar growth-inhibitory results were obtained (data not shown).

The effect of Ang-1 on tumour neovascularisation and growth is controversial. The concept that the angiopoietins are important mediators of tumour growth was elegantly demonstrated by Holash and associates, who demonstrated the importance of the coordinated induction of the angiopoietins and VEGF in tumour angiogenesis (Holash *et al*, 1999). Other studies have suggested that Ang-1 may be pro-angiogenic. Shim *et al* (2001) recently demonstrated that overexpression of antisense Ang-1 mRNA by HeLa cells inhibited angiogenesis and xenografted tumour growth in immunodeficient mice. An increased neovascularisation effect by Ang-1 overexpression was also described by Suri *et al* (1998) using a transgenic mouse model. These authors concluded that Ang-1 could be used in combination with VEGF to promote therapeutic angiogenesis.

The importance of the cooperation of Ang-1 and VEGF in induction of angiogenesis has been demonstrated in several malignant and non-malignant models of angiogenesis (Peters, 1998; Ray *et al*, 2000). In a transgenic mouse model with increased co-expression of Ang-1 and VEGF, angiogenesis was also increased (Thurston *et al*, 1999). This phenomenon of an enhanced vasculogenesis by combining Ang-1 with VEGF was also demonstrated by Chae *et al* (2000). Using a rabbit ischaemic hind-limb model, they demonstrated that a combination of Ang-1 and VEGF gene delivery resulted in the formation of larger blood vessels, increased blood flow and higher capillary density than was seen when either factor was delivered alone.

In contrast, several studies suggest that Ang-1 may inhibit tumour angiogenesis. In our previous studies, we were able to demonstrate that imbalances in Ang expression may regulate growth and angiogenesis of human colon cancer (Ahmad *et al*, 2001). In a subcutaneous xenografted colon cancer model, differential expression of angiopoietins affected both tumour growth and angiogenesis. Ang-1 overexpression significantly inhibited tumour growth and angiogenesis in this model (Ahmad *et al*, 2001). These findings are now supported by the results of a recent study by Tian *et al* (2002), who found that Ang-1 overexpression by MCF-7 breast cancer cells resulted in stabilisation of tumour edge-associated blood vessels. In addition, tumour cell proliferation decreased significantly in the presence of Ang-1, resulting in a reduced xenografted tumour growth. Tie2 receptor was found to be present in vascular smooth muscle cells in culture in addition to endothelial cells. On the basis of these results, Tian *et al* (2002) concluded that vascular stabilisation by Ang-1 accounts for the inhibition of tumour growth. In a previous study, Hayes *et al* (2000) also demonstrated that Ang-1 overexpression in MCF-7 human breast cancer cells caused a significant retardation in tumour growth despite the high co-expression of a potent angiogenic growth factor (fibroblast growth factor-1).

The results of our present study suggest that the effects of Ang-1 on tumour growth were mediated by inhibition of tumour angiogenesis and that the abrogation of ascites formation was due to a reduction of vascular permeability (plasma leakage). In this model, Ang-1 overexpression did not prevent peritoneal implantation of tumours, although peritoneal nodules were fewer and smaller in the Ang-1 group. Therefore, Ang-1 prevented angiogenesis dependent tumour outgrowth by inhibiting neovascularisation, which was reflected by measurement of the largest tumour in mice.

As demonstrated in our permeability assay, Ang-1 levels in CM from Ang-1-transfected cells abrogated the increase of plasma leakage (dye leakage) of dermal microvasculature caused by tumour cell–derived growth factors (KM12L4 cells express relatively high amounts of VEGF (Ellis *et al*, 1996)), suggesting that Ang-1 is an important mediator of vascular stabilisation and permeability and may override VEGF-mediated vessel leakage. Other factors might have been present in conventional medium or conditioned medium that was used in this assay that potentially might have affected vascular permeability. However, VEGF is known as the strongest inducer of vascular leakage (Dvorak, 2000; Dvorak *et al*, 1999). Therefore, although possible, it is unlikely that ingredients of conventional medium and factors besides VEGF played a significant role in this assay. This hypothesis is supported by the results of other groups who have investigated the effects of Ang-1 on vascular permeability and vessel stabilisation. Gamble *et al* (2000) showed that administration of recombinant Ang-1 supported the localisation of a cell adhesion molecule (PECAM-1) into junctions between endothelial cells, strengthening these junctions. In addition, basal permeability of endothelial cell monolayers was reduced in a dose-dependent manner by treatment with recombinant Ang-1, and endothelial cell permeability responses to permeability-inducing agents such as thrombin and VEGF were inhibited by treatment with Ang-1. Thurston *et al* (1999) have reported anti-permeability properties of Ang-1 in two different studies evaluating the impact of VEGF on plasma leakage of adult vasculature and in a previous experiment using a transgenic mouse model overexpressing both Ang-1 and VEGF. In the latter study, co-expression of Ang-1 and VEGF had an additive effect on angiogenesis but resulted in the formation of leakage-resistant vessels. Furthermore, the authors showed that acute administration of Ang-1 protected adult vasculature from leakage mediated by VEGF and inflammatory cytokines (Thurston *et al*, 2000).

In conclusion, our study indicates that Ang-1 was an important mediator of tumour angiogenesis and vascular permeability in an experimental model of peritoneal carcinomatosis from human colon cancer. The inhibition of vascular permeability by Ang-1 is especially relevant in a tumour system associated with a great deal of morbidity due to ascites formation that accompanies carcinomatosis. Our studies suggest that Ang-1 could potentially serve as an anti-angiogenic/anti-permeability agent in the treatment of carcinomatosis from colorectal cancer.
